# Quantitative Whole-Leg Radiographic Assessment of Coronal Knee–Ankle Alignment: Associations with Talar Inclination and Exploratory Changes After Knee Osteotomy

**DOI:** 10.3390/diagnostics16193165

**Published:** 2026-09-29

**Authors:** Ralf Henkelmann, Anna Isabel Müllenbach, Timm Denecke, Pierre Hepp, Jeanette Henkelmann

**Affiliations:** 1 Department for Orthopedics, Trauma and Plastic Surgery, University Hospital Leipzig, 04103 Leipzig, Germany; 2 Department for Diagnostic and Interventional Radiology, University Hospital Leipzig, Liebigstraße 20, 04103 Leipzig, Germany

**Keywords:** whole-leg radiography, quantitative imaging, lower-limb alignment, knee–ankle joint line angle, talar inclination, high tibial osteotomy, distal femoral osteotomy

## Abstract

**Background/Objectives:** Standing whole-leg radiographs are central to coronal deformity assessment, but mechanical axis deviation (MAD) may not fully characterize the orientation of individual joint surfaces. We evaluated ankle joint orientation across coronal alignment phenotypes and, secondarily, explored descriptive changes after knee realignment osteotomy. **Methods:** This retrospective single-center study included 382 whole-leg radiographs from 374 patients and an exploratory longitudinal subgroup of 80 limbs from 75 patients before and after high tibial osteotomy (HTO) or distal femoral osteotomy (DFO). Limbs were classified as varus, valgus, or near-neutral by MAD. Talar inclination was the primary imaging outcome; knee–ankle joint line angle (KAJA), talar tilt, and segmental alignment parameters were assessed by three independent readers. **Results:** Talar inclination was greater in valgus than varus or near-neutral limbs (10.5 ± 5.9° vs. 4.7 ± 3.3° and 4.7 ± 3.5°; *p* < 0.001). Inter-reader reliability was excellent (ICC 0.91–0.96). MAD correlated with talar inclination (ρ = −0.426, *p* < 0.001), while KAJA showed a larger univariable correlation (ρ = 0.611, *p* < 0.001). Adding KAJA to age, sex, and MAD increased model R^2^ from 0.237 to 0.551; because these measures are geometrically interrelated, this increment was interpreted as descriptive model fit rather than an independent biological contribution. In the exploratory postoperative subgroup, talar inclination decreased in valgus limbs (−3.2 ± 5.2°, *p* = 0.012), whereas changes were not significantly associated with the magnitude of MAD correction. **Conclusions:** Whole-leg radiography demonstrates distinct knee–ankle alignment patterns not captured by MAD alone. KAJA provides complementary descriptive morphologic information regarding talar orientation, but should not be interpreted as an independent biological predictor. Postoperative subgroup findings are hypothesis-generating because of small, unbalanced groups and procedure-related confounding; no clinical outcome or three-dimensional hindfoot inference can be drawn from these data.

## 1. Introduction

Coronal lower-limb alignment is an important determinant of load distribution across the lower extremity and is associated with the development and progression of compartment-specific knee osteoarthritis (OA) [1,2,3,4,5,6]. Consequently, radiographic assessment of lower-limb alignment is a central component of diagnostic work-up and preoperative planning in patients with symptomatic malalignment and unicompartmental knee degeneration.

High tibial osteotomy (HTO) and distal femoral osteotomy (DFO) are established joint-preserving procedures that redistribute joint loading by correcting coronal deformity [7,8,9,10]. Although planning has traditionally focused on the knee and the mechanical axis, the ankle and hindfoot form part of the same weight-bearing chain. Previous studies have demonstrated associations between lower-limb malalignment and distal joint orientation and have reported changes in ankle alignment after realignment around the knee [10,11,12,13,14,15,16,17,18,19]. These observations are clinically relevant because a correction that is appropriate at the knee may alter distal joint orientation in a way that is not captured by the mechanical axis alone.

Recent work by Maione et al. evaluated 60 knees (37 lateral closing-wedge HTOs and 23 medial closing-wedge DFOs) and demonstrated significant postoperative changes in tibio-talar inclination and Mikulicz lateral distal tibial angle, with larger tibio-talar changes after DFO [14]. Contemporary HTO studies, including Oh et al. [16], Oktem et al. [12], and Dorst et al. [18], likewise demonstrated postoperative changes in ankle alignment. Thus, the novelty of the present study is not the observation that knee osteotomy can alter ankle alignment. Rather, we examine ankle orientation across a broader cross-sectional spectrum of varus, valgus, and near-neutral phenotypes and assess whether the joint-line relationship captured by KAJA provides descriptive morphologic information beyond overall axis deviation.

From a quantitative imaging perspective, MAD provides a global measure of coronal alignment but does not directly describe the orientation of individual joint surfaces. The knee–ankle joint line angle (KAJA), defined by the relationship between the proximal and distal tibial joint lines, may capture complementary morphologic information within the weight-bearing chain [17]. Because alignment parameters are anatomically interrelated, however, their associations should not be interpreted as evidence that one parameter represents an independent mechanistic determinant.

The primary aim of this study was to quantitatively characterize the association between coronal lower-limb alignment and ankle joint orientation on standing whole-leg radiographs and to determine whether talar inclination differs between varus, valgus, and near-neutral alignment phenotypes. The central new question was whether baseline coronal phenotype and KAJA identify distinct two-dimensional ankle-orientation patterns not captured by MAD alone. The secondary aim was to explore pre- to postoperative changes in ankle joint orientation after HTO or DFO and their relationship to changes in mechanical axis and joint-line orientation. We hypothesized that valgus-aligned limbs would demonstrate greater talar inclination than varus and near-neutral limbs and that joint-line orientation would provide complementary imaging information to MAD. The postoperative analysis was considered exploratory and was not intended to establish a causal adaptation mechanism.

## 2. Materials and Methods

### 2.1. Study Design and Patient Selection

This retrospective, single-center observational study consisted of (1) a cross-sectional analysis of the association between coronal lower-limb alignment and ankle joint orientation and (2) an exploratory pre- to postoperative subgroup analysis evaluating changes after surgical realignment around the knee. The study was approved by the Ethics Committee of the Medical Faculty of Leipzig University (approval no. 185/25-ek) and conducted in accordance with the Declaration of Helsinki. Informed consent was waived according to local regulations.

Patients who underwent standing whole-leg radiography at a tertiary referral center between 2014 and 2025 were screened. Radiographs had been obtained for clinical indications including lower-limb malalignment, osteoarthritis, limb-length discrepancy, and preoperative planning. Each eligible limb was treated as one radiographic case; eight patients contributed bilateral examinations. The primary analyses used limb-level observations without cluster adjustment. To assess whether this non-independence materially affected the findings, all primary cross-sectional analyses were repeated in a sensitivity dataset containing one limb per patient.

Inclusion criteria were full-length weight-bearing radiographs including the hip, knee, and ankle; full knee extension; and standardized rotational positioning with central patellar alignment, symmetric femoral condyles, and physiologic tibiofibular overlap [20,21,22]. Radiographs that did not meet predefined quality criteria, as well as limbs with previous joint arthroplasty, fractures, shaft deformities, or structural deformities affecting the lower extremity, were excluded. For the longitudinal subgroup, postoperative standing whole-leg radiographs obtained at least 6 weeks after surgery were required to ensure full weight-bearing conditions.

The level of corrective osteotomy was determined by the anatomical location of the osseous deformity in every case. Tibial deformities were treated by HTO, whereas femoral deformities were treated by DFO. Thus, the choice between HTO and DFO was based on deformity localization rather than on the overall magnitude or direction of MAD. The choice of an opening- or closing-wedge technique was subsequently determined by the direction of the deformity (varus or valgus) at the respective anatomical level. Accordingly, limbs classified as near-neutral according to MAD could still undergo corrective osteotomy when clinically relevant segmental osseous malalignment was present.

### 2.2. Radiographic Acquisition and Quantitative Assessment

Radiographic measurements were performed on standardized standing whole-leg radiographs using mediCAD 2D (version 7.0.0.3, Hectec GmbH, Landshut, Germany) and IDS7 (version 25.1, Sectra Workstation, Linköping, Sweden). The measurement strategy combined a global axis measure (MAD), segmental osseous alignment parameters, and direct joint-orientation measures at the knee and ankle.

MAD was used to characterize overall coronal limb alignment (Table 1). Based on established deformity-analysis principles, limbs were categorized as varus (MAD > 15 mm medial), valgus (MAD > 10 mm lateral), or near-neutral between these thresholds [23,24]. These asymmetric cutoffs reflect the established clinical MAD convention, in which the physiological mechanical axis is slightly medial to the knee center; they were prespecified and were not derived from the study outcome data. In the dataset, medial deviation was represented by positive MAD values and lateral deviation by negative values. The primary outcome was talar inclination, defined as the angle between a tangent to the talar dome and a horizontal ground reference line parallel to the horizontal image axis/floor during standardized standing acquisition [15,25]. As an absolute two-dimensional image-plane measure referenced to horizontal, talar inclination was interpreted as a radiographic orientation parameter rather than a direct measure of subtalar or hindfoot compensation. Talar tilt was defined as the angle between the tibial plafond and the talar dome. KAJA was defined as the angle between lines tangent to the articular surfaces of the proximal tibia and distal tibia; a positive value indicates a valgus relationship and a negative value a varus relationship [17]. Additional measurements included mMPTA, mLDFA, mLDTA, and JLCA according to standard deformity-analysis principles [4,23,24].

All study-specific ankle measurements were performed independently by three readers: a radiological researcher (A.I.M.), a board-certified musculoskeletal radiologist (J.H.), and an orthopaedic surgeon specialized in knee surgery (R.H.). For the statistical analyses, the arithmetic mean of the three reader measurements was used for talar inclination, talar tilt, and KAJA. Readers were blinded to clinical information and surgical details. Complete blinding to pre- versus postoperative status was not possible because osteosynthesis hardware was visible on postoperative radiographs.

### 2.3. Reliability Analysis

Inter-reader reliability was assessed for talar inclination, talar tilt, and KAJA using intraclass correlation coefficients (ICC) based on a two-way random-effects model with absolute agreement. ICCs are reported with 95% confidence intervals and were interpreted according to Koo and Li, with values >0.90 considered excellent [26]. Established lower-limb alignment measurements (including MAD, mMPTA, mLDFA, and mLDTA) were not reassessed because excellent interobserver reliability has previously been reported for digital deformity analysis [27].

### 2.4. Statistical Analysis

Descriptive statistics were calculated for all variables. Normality was assessed visually using histograms and Q–Q plots and statistically using the Shapiro–Wilk test. For the cross-sectional analysis, differences between varus, valgus, and near-neutral alignment groups were analyzed using one-way ANOVA or Welch’s ANOVA when homogeneity of variance was violated. Post hoc comparisons were performed using Tukey or Games–Howell tests as appropriate. Effect sizes were reported as eta squared (η^2^). Spearman rank correlation coefficients were used to assess associations between alignment measures and ankle orientation.

To avoid unstable inference from highly correlated alignment variables, multivariable analysis was restricted to a parsimonious hierarchical linear regression addressing the prespecified imaging question of whether KAJA adds explanatory information beyond age, sex, and MAD. Model 1 included age, sex, and MAD; Model 2 additionally included KAJA. The change in explained variance (ΔR^2^) was used only to describe incremental model fit. Because KAJA and the other alignment measures are geometrically interrelated, ΔR^2^ was not interpreted as unique biological variance attributable to KAJA or as evidence of an independent predictor. Regression coefficients were interpreted as associations rather than independent causal effects. A broader exploratory model containing MAD, KAJA, mMPTA, mLDFA, mLDTA, and JLCA simultaneously demonstrated severe multicollinearity (VIF up to 41.78) and was therefore not used to infer or rank the independent importance of individual alignment parameters.

For the exploratory longitudinal subgroup, pre- to postoperative differences were assessed using paired *t*-tests or Wilcoxon signed-rank tests depending on data distribution. Between-group differences in change scores were assessed using ANOVA, and correlations between change scores were evaluated. Because the surgical subgroups were small and unbalanced and because procedure type was associated with preoperative alignment phenotype, no causal interpretation was assigned to subgroup-specific changes. The longitudinal analysis was not covered by the a priori power calculation and was considered hypothesis-generating.

To evaluate possible non-independence arising from bilateral radiographs, a sensitivity analysis excluded one limb from patients with bilateral examinations. All primary cross-sectional analyses were repeated in the reduced dataset. Statistical tests were two-sided and *p* < 0.05 was considered significant. Analyses were performed using JASP (version 0.95.4; University of Amsterdam, Amsterdam, The Netherlands). The a priori sample size calculation for the primary cross-sectional one-way ANOVA assumed Cohen’s f = 0.20, α = 0.05, and 80% power, yielding a minimum required sample of 246 cases.

## 3. Results

### 3.1. Cohort Characteristics and Reliability

The cross-sectional cohort comprised 374 patients with 382 standing whole-leg radiographs: 102 varus, 116 valgus, and 164 near-neutral limbs according to MAD (Table 2). The exploratory longitudinal cohort comprised 75 patients with 80 limbs (43 varus, 20 valgus, and 17 near-neutral) with paired pre- and postoperative radiographs after HTO or DFO. Postoperative standing whole-leg radiographs were obtained at a median of 38.0 weeks (IQR 14.1–75.8) after surgery. The inclusion process is shown in Figure 1.

Inter-reader reliability was excellent for all study-specific ankle orientation parameters: ICC 0.94 (95% CI 0.92–0.96) for talar inclination, 0.91 (95% CI 0.88–0.94) for talar tilt, and 0.96 (95% CI 0.94–0.97) for KAJA.

### 3.2. Coronal Alignment Phenotype and Ankle Joint Orientation

Talar inclination differed significantly between varus, valgus, and near-neutral groups (Welch ANOVA: F = 49.0, *p* < 0.001, η^2^ = 0.278). Valgus-aligned limbs demonstrated greater talar inclination than both varus and near-neutral limbs (10.5 ± 5.9° vs. 4.7 ± 3.3° and 4.7 ± 3.5°, respectively; Figure 2). In contrast, talar tilt did not differ between groups. Segmental osseous parameters mMPTA, mLDFA, and mLDTA also differed significantly across alignment groups (Table 3).

### 3.3. Association of MAD and KAJA with Talar Inclination

MAD showed a moderate negative correlation with talar inclination (Spearman ρ = −0.426, *p* < 0.001), whereas KAJA showed a larger positive univariable correlation (ρ = 0.611, *p* < 0.001). In the hierarchical regression, Model 1 (age, sex, and MAD) explained 23.7% of the variance in talar inclination (R^2^ = 0.237). Addition of KAJA increased explained variance to 55.1% (R^2^ = 0.551; ΔR^2^ = 0.314). In the combined model, both KAJA (β = 0.579, *p* < 0.001) and MAD (β = −0.346, *p* < 0.001) remained associated with talar inclination. Because these measures describe related components of coronal alignment, the coefficients were interpreted as complementary associations rather than evidence of biological or diagnostic superiority.

An exploratory interaction model including KAJA and alignment group yielded R^2^ = 0.618 and suggested that the association between KAJA and talar inclination varied across alignment phenotypes, with steeper fitted relationships in valgus and near-neutral limbs than in varus limbs (Figure 3). This interaction was interpreted descriptively and not as evidence of an alignment-specific causal compensation mechanism.

### 3.4. Exploratory Longitudinal Changes After Knee Osteotomy

In the longitudinal subgroup, talar inclination decreased in valgus-aligned limbs from 10.2 ± 5.2° preoperatively to 7.0 ± 5.4° postoperatively (mean change −3.2 ± 5.2°, *p* = 0.012). No statistically significant pre- to postoperative change in talar inclination was observed in varus or near-neutral limbs. Between-group differences in talar inclination change were significant (F(2,77) = 4.67, *p* = 0.012), with larger reductions in the valgus group than in the varus (*p* = 0.015) and near-neutral groups (*p* = 0.040). No significant between-group differences were observed for changes in talar tilt, KAJA, or mLDTA (Table 4).

Changes in KAJA were moderately correlated with changes in talar inclination (r = 0.409, *p* < 0.001), whereas the magnitude of MAD correction was not significantly associated with talar inclination change. The valgus group had the highest baseline talar inclination and differed in demographic characteristics and deformity location, with valgus deformities predominantly corresponding to femoral correction (Figure 4). These findings may therefore reflect baseline-dependent change, regression to the mean, osteotomy-level distribution, or true morphologic differences; the present dataset cannot separate these explanations.

### 3.5. Sensitivity Analysis

Excluding one limb from patients with bilateral radiographs yielded essentially unchanged primary cross-sectional results. The direction, magnitude, and statistical significance of the principal associations were preserved, supporting the robustness of the cross-sectional findings to bilateral inclusion.

## 4. Discussion

The principal finding of this study is that ankle joint orientation on standing whole-leg radiographs differs across coronal lower-limb alignment phenotypes. Valgus-aligned limbs demonstrated substantially greater talar inclination than varus and near-neutral limbs, whereas talar tilt remained similar across groups. The absence of a group difference in talar tilt suggests that the observed phenotype differences primarily concern the absolute orientation of the talar dome relative to the ground reference rather than a detectable difference in coronal tibiotalar congruity on these two-dimensional radiographs. Both MAD and KAJA were associated with talar inclination, and adding KAJA to a model containing age, sex, and MAD increased descriptive model fit. The key contribution is therefore the cross-sectional demonstration that global mechanical axis position and knee-to-ankle joint-line orientation provide complementary descriptive imaging information rather than interchangeable measures of the same radiographic feature.

The relationship between KAJA and talar inclination is particularly relevant for radiographic deformity assessment. MAD describes where the mechanical axis crosses the knee but does not directly characterize the orientation of individual joint surfaces, whereas KAJA represents the angular relationship between the proximal and distal tibial joint lines [17]. In the present cohort, adding KAJA to age, sex, and MAD improved descriptive model fit. This increment should not be interpreted as an independent biological contribution because talar inclination and KAJA share related joint-line geometry. The broader exploratory model confirmed substantial multicollinearity among alignment parameters (VIF up to 41.78), precluding ranking of individual coefficients as independent biological or diagnostic determinants. Accordingly, direct joint-line assessment should be viewed as complementary to, rather than a replacement for, global axis assessment.

The near-neutral group further illustrates why an axis-centered interpretation alone may be incomplete. Limbs with a near-neutral MAD can still have clinically relevant segmental osseous deformity at the tibial or femoral level. In the surgical subgroup, the level of osteotomy was determined by the anatomical location of the deformity: tibial deformities were treated with HTO and femoral deformities with DFO, while opening- versus closing-wedge technique was selected according to the varus or valgus direction at that level. Thus, a near-neutral mechanical axis does not necessarily imply neutral segmental morphology, and interpretation of standing whole-leg radiographs should integrate global alignment, segmental bone angles, and distal joint orientation.

These findings are consistent with previous reports that ankle alignment may change after knee osteotomy, including the studies by Maione et al. [14], Oh et al. [16], Oktem et al. [12], and Dorst et al. [18], as well as recent systematic reviews [7,10]. Accordingly, postoperative ankle reorientation itself is not presented here as novel. The primary contribution is the larger cross-sectional comparison across varus, valgus, and near-neutral phenotypes and the joint consideration of MAD and KAJA, demonstrating that baseline phenotype and joint-line morphology are already associated with marked differences in talar orientation before surgery. The greater postoperative change observed in our valgus subgroup is directionally consistent with the larger changes reported after DFO, but valgus phenotype and femoral deformity were closely linked in our cohort and cannot be separated statistically.

The longitudinal findings require cautious interpretation. Talar inclination decreased significantly only in the valgus subgroup, and changes in KAJA correlated with changes in talar inclination, whereas the magnitude of MAD correction was not significantly associated with talar inclination change. Because the surgical groups were small and unbalanced and differed in age, sex distribution, baseline talar inclination, and deformity location—with valgus deformities predominantly corresponding to femoral correction—baseline-dependent change, regression to the mean, and osteotomy level cannot be separated. The longitudinal analysis should therefore be regarded as descriptive and hypothesis-generating and does not establish phenotype-specific, osteotomy-level, or causal adaptation effects. From an imaging perspective, the excellent inter-reader reliability of talar inclination, talar tilt, and KAJA (ICC 0.91–0.96) supports the reproducibility of these measurements on standardized standing whole-leg radiographs.

For clinical imaging, the practical implication is to inspect the ankle, distal tibial joint line, and knee-to-ankle joint-line relationship when full-length weight-bearing radiographs are obtained for knee deformity or osteotomy planning, rather than relying on MAD alone. Patients with similar MAD values may have different joint-line relationships, and postoperative ankle orientation cannot be inferred solely from the magnitude of mechanical axis correction. These radiographic findings do not support changing an osteotomy target, do not establish that a specific ankle orientation causes symptoms, and do not predict clinical outcome; patient-reported or functional outcome measures were not available. The study is limited by its retrospective single-center design, heterogeneous clinical indications, broad age dispersion in the valgus cohort, and two-dimensional static measurements. Talar inclination is projection-dependent and does not provide a three-dimensional assessment of the talus, subtalar joint, or hindfoot. Dedicated hindfoot alignment views (e.g., Saltzman view) and weight-bearing CT were not available [10,13,28]; consequently, the present data cannot distinguish fixed osseous morphology from rotational projection effects, soft-tissue behavior, or subtalar/hindfoot compensation. The primary limb-level regression models did not account for within-patient clustering of bilateral radiographs, although the one-limb-per-patient sensitivity analysis yielded comparable results. Prospective studies integrating standardized hindfoot imaging, clinical outcomes, and uniform postoperative intervals are needed to determine the clinical consequences of these imaging patterns.

## 5. Conclusions

Standing whole-leg radiographs demonstrate clear differences in ankle joint orientation across coronal lower-limb alignment phenotypes. Valgus-aligned limbs show greater talar inclination than varus and near-neutral limbs, and KAJA provides complementary descriptive morphologic information to MAD when characterizing two-dimensional knee–ankle alignment. Postoperative changes after HTO or DFO were heterogeneous and were not proportional to the magnitude of mechanical axis correction in this exploratory subgroup. These findings support a multi-parameter radiographic description that integrates global mechanical alignment, segmental bone geometry, and distal joint-line orientation. They do not establish clinical relevance, symptom correlation, independent biological effects, or three-dimensional hindfoot compensation; the postoperative observations remain hypothesis-generating and require prospective confirmation.

## Figures and Tables

**Figure 1:**
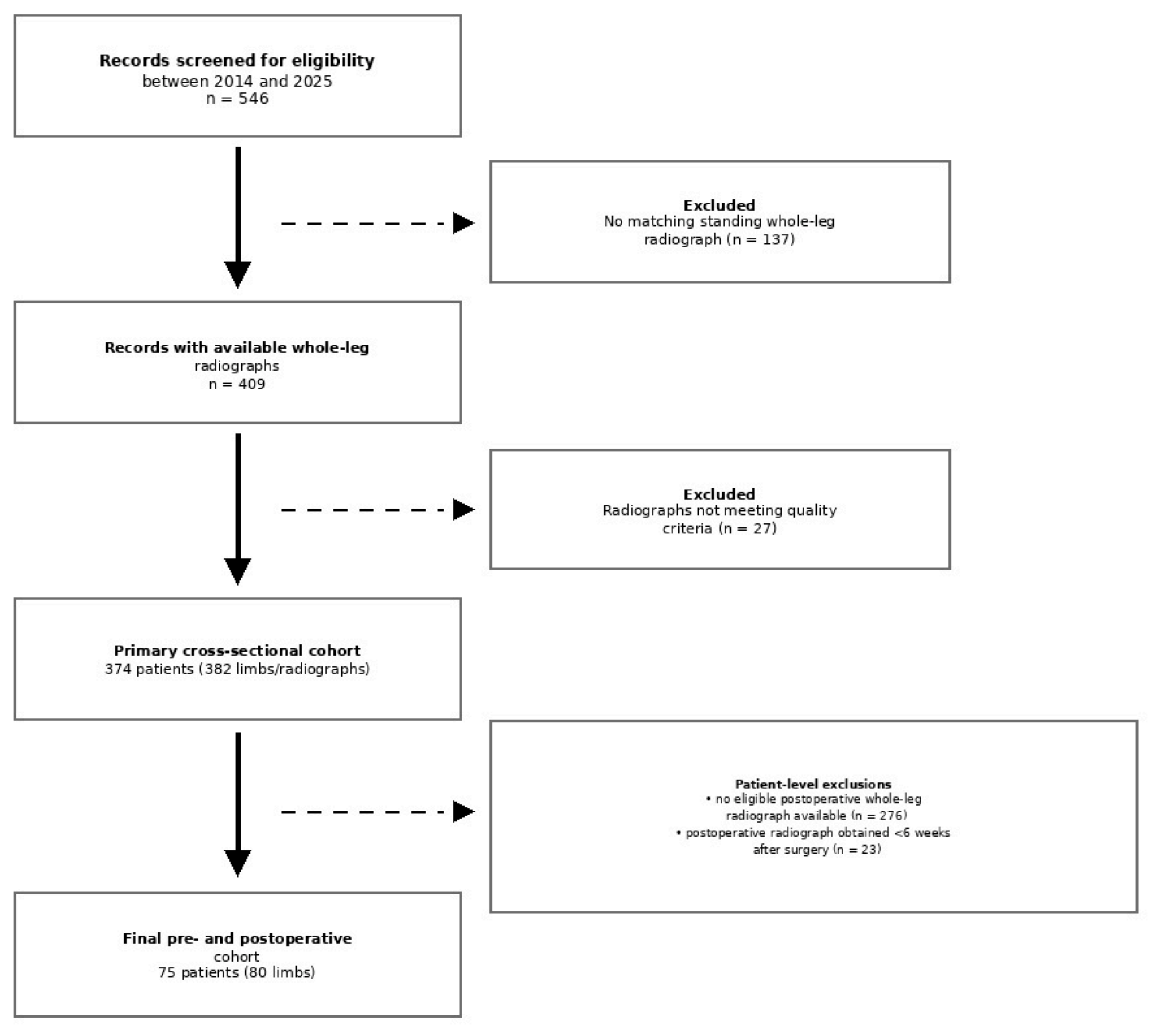
Flowchart of study inclusion for the cross-sectional and exploratory pre- to postoperative analyses. Final analytic cohorts are reported as patients and limbs.

**Figure 2:**
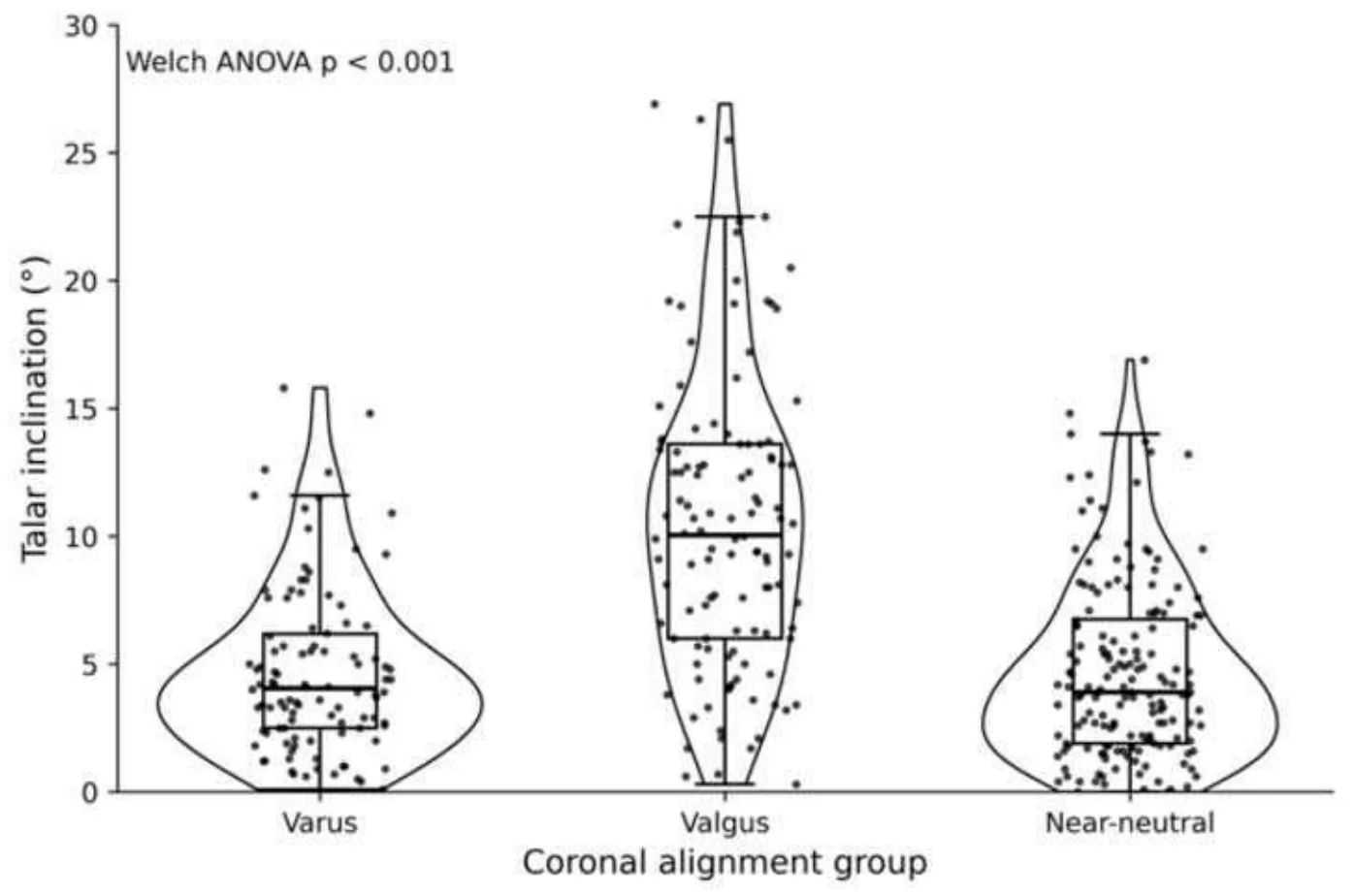
Distribution of talar inclination across varus, valgus, and near-neutral alignment groups. The violin plots show the distribution with individual observations and boxplots; Welch ANOVA *p* < 0.001. Dots represent individual observations, and the embedded boxplots indicate the median and interquartile range.

**Figure 3:**
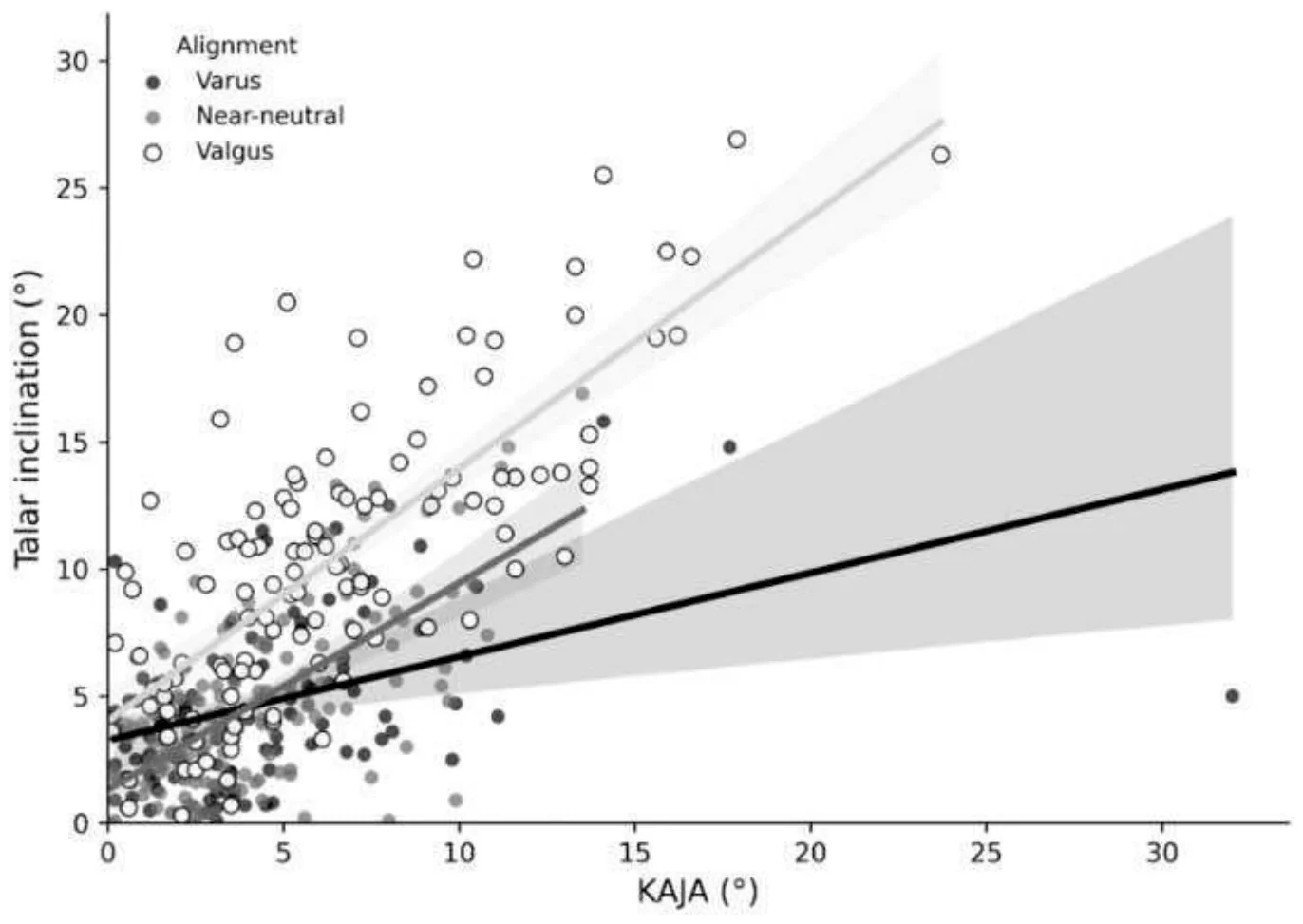
Association between knee–ankle joint line angle (KAJA) and talar inclination according to coronal alignment group. Lines show group-specific fitted relationships; shaded areas indicate 95% confidence intervals.

**Figure 4:**
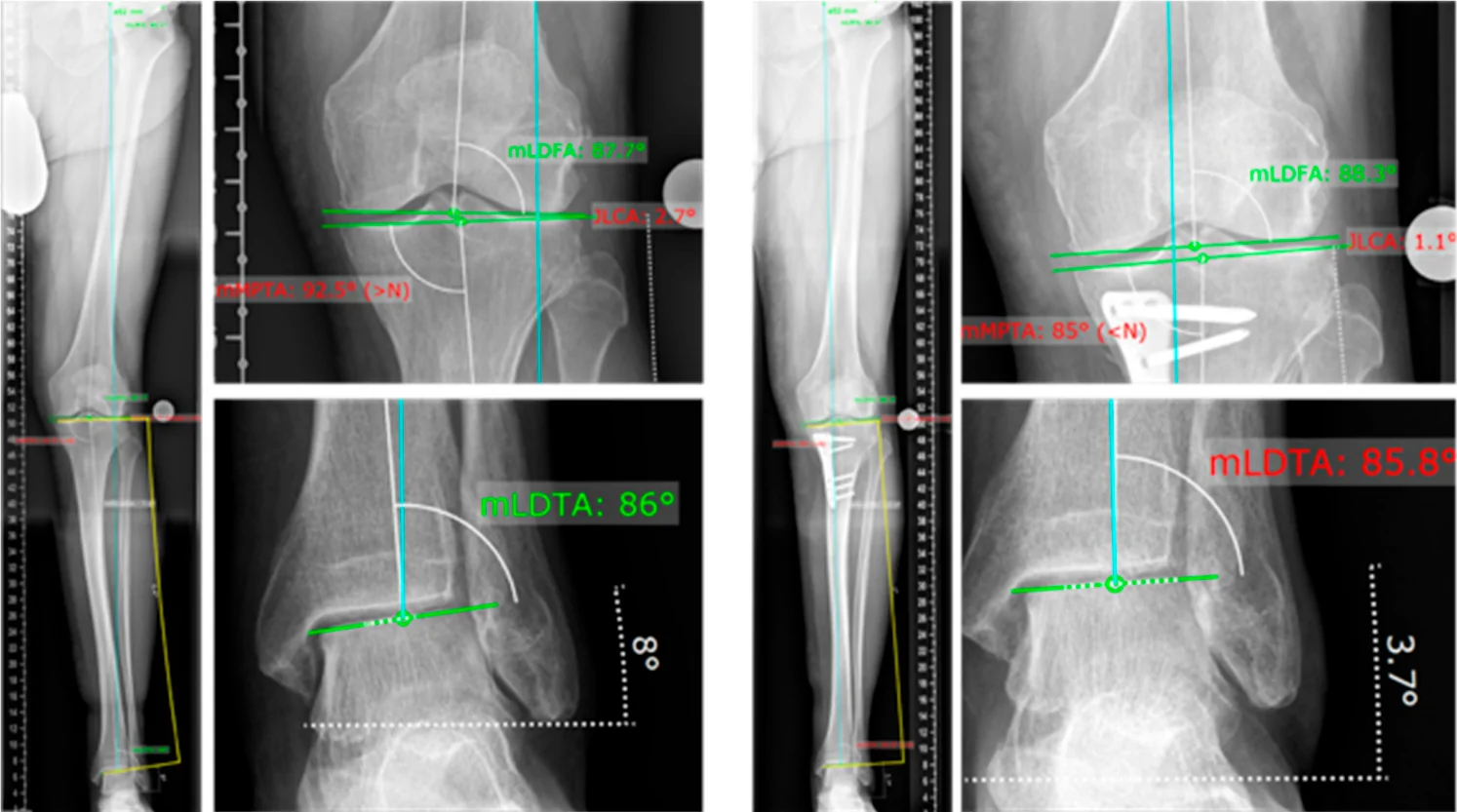
Representative valgus case demonstrating change in ankle joint orientation after corrective osteotomy. Preoperative and postoperative standing radiographs illustrate the relationship between lower-limb alignment and distal joint orientation. This example is illustrative and does not establish a uniform mechanism of postoperative change.

**Table 1:** Radiographic parameters used for quantitative lower-limb assessment.

Parameter	Definition/Reference Lines	Primary Imaging Information	Sign Convention/Interpretation
MAD	Offset of the knee center relative to the hip-to-ankle mechanical axis	Global coronal limb alignment	Medial deviation positive (varus); lateral deviation negative (valgus)
Talar inclination	Angle between the tangent to the talar dome and the horizontal ground reference	Absolute orientation of the talar dome under weight-bearing	Degrees; higher values indicate greater coronal inclination
Talar tilt	Angle between the tibial plafond and talar dome	Congruity/relative tibiotalar joint orientation	Degrees
KAJA	Angle between tangents to the proximal tibial and distal tibial articular surfaces	Relative knee-to-ankle joint-line orientation	Positive = valgus relationship; negative = varus relationship [17]
mMPTA	Medial angle between tibial mechanical axis and proximal tibial joint line	Proximal tibial contribution to coronal deformity	Degrees
mLDFA	Lateral angle between femoral mechanical axis and distal femoral joint line	Distal femoral contribution to coronal deformity	Degrees
mLDTA	Lateral angle between tibial mechanical axis and distal tibial joint line	Distal tibial orientation	Degrees
JLCA	Angle between distal femoral and proximal tibial joint lines	Intra-articular/soft-tissue contribution to coronal alignment	Degrees

**Table 2:** Baseline characteristics of the cross-sectional cohort and surgical subgroup according to coronal alignment group.

Variable	Varus	Valgus	Near-Neutral	*p* Value
Cross-sectional cohort	*n* = 102	*n* = 116	*n* = 164	
Age (years)	51.8 ± 13.5	51.4 ± 23.1	43.2 ± 16.5	<0.001
Sex, male *n* (%)	83 (81.4)	29 (25.0)	76 (46.3)	<0.001
MAD (mm)	28.4 ± 12.2	−25.5 ± 14.2	1.3 ± 7.1	—
Surgical subgroup	*n* = 43	*n* = 20	*n* = 17	
Age (years)	48.2 ± 8.4	31.9 ± 11.9	44.9 ± 10.8	<0.001
Sex, male *n* (%)	35 (81.4)	6 (30.0)	12 (70.6)	<0.001
Preoperative MAD (mm)	28.1 ± 11.7	−20.8 ± 11.2	7.0 ± 8.5	—

**Table 3:** Radiographic parameters according to coronal alignment in the cross-sectional cohort.

Parameter	Varus	Valgus	Near-Neutral	*p* Value	η^2^
Talar inclination (°)	4.7 ± 3.3	10.5 ± 5.9	4.7 ± 3.5	<0.001	0.278
Talar tilt (°)	1.2 ± 1.1	1.2 ± 1.0	1.2 ± 1.0	0.99	<0.001
KAJA (°)	4.5 ± 4.3	6.5 ± 4.5	4.2 ± 2.8	<0.001	0.070
mMPTA (°)	85.1 ± 2.7	91.2 ± 2.7	88.3 ± 2.6	<0.001	0.426
mLDFA (°)	89.7 ± 2.0	85.7 ± 2.4	88.0 ± 2.2	<0.001	0.326
mLDTA (°)	87.4 ± 4.4	84.9 ± 5.2	86.8 ± 4.4	<0.001	0.047

**Table 4:** Exploratory pre- to postoperative changes in ankle alignment parameters following corrective osteotomy.

Parameter	Varus (*n* = 43)	Valgus (*n* = 20)	Near-Neutral (*n* = 17)	Between-Group *p*
Δ Talar inclination (°)	0.1 ± 4.3	−3.2 ± 5.2	0.3 ± 2.7	0.012
Δ Talar tilt (°)	0.0 ± 1.0	−0.1 ± 0.8	0.2 ± 0.5	0.494
Δ KAJA (°)	1.0 ± 4.5	−0.8 ± 3.3	0.3 ± 3.3	0.266
Δ mLDTA (°)	0.5 ± 3.3	0.1 ± 3.6	−0.9 ± 2.2	0.270

## Data Availability

The datasets analyzed during the current study contain patient-related radiographic data and are not publicly available. Data may be available from the corresponding author on reasonable request and subject to applicable institutional and data-protection requirements.
